# Abstracts from Foreign Journals

**Published:** 1898-11

**Authors:** J. Preston Hoskins

**Affiliations:** Princeton, N. J.


### ABSTRACTS FROM FOREIGN JOURNALS.

### GERMAN.

Under the Direction of J. Preston Hoskins, Ph.D.,
PRINCETON, N. J.

Comparative Statistics of Cysticercus Cellulosæ in the Eye of Man and of Animals. (By Meat-inspector Prettner, Prague.)—According to Leuckart, the favorite places for cyst. cell. are the muscles of the breast and shoulders, the tongue, the diaphragm, and the thighs. The heart and underskin also are often covered with blotches, as well as the nerve-centres and eyes, while the intestines as a whole are seldom affected.

Von Graefe observed about one hundred cases of these pimples in the deeper parts of the human eye, and reckons the frequency of the cysticercus for the Berlin eye-clinic at one in every thousand cases of eye trouble.

In the front part of the eye, on the other hand, he noted only nine cases in all: five under the conjunctiva, two in the front chamber, one in the lens, and one situated in the orbita—in all, hardly one-eighth of those which occur in the subretinal tissues. In all the cases observed by von Graefe there was only a single cysticercus in the eye. This fact is confirmed by O. Becker, who in only one case found more than one cysticercus in the human eye. In strong contrast, a considerable number have occasionally been found in the eyes of swine. Nordmann, in one case, counted twelve, of which six were in the ball, and the others between the sclerotica and the chorioïdea.

The first man who ever observed this pimple is supposed to have been Adrian von der Spiegel, about two hundred and seventy years ago. But it is questionable whether it was a case of cyst. cell. Not until the present century was the cyst. cell. recognized in the human eye.

Von Schott, in the year 1830, found a living bladder-worm (?) in the eye of a man, and by an operation in the cornea extracted it. Many others report cases of pimples in the eye, and in every case it is a question of the swine-pimple. Prof. Hirschberg, between 1869 and 1885, found seventy cases of cyst. cell. among 60,000 cases of eye trouble. In recent years reports have been much more seldom. Between 1886 and 1889 Hirschberg, in 30,-

000 cases of eye trouble, observed only one case which was operated upon. This striking rarity cannot be accidental, and Hirschberg is inclined to attribute it to the stricter system of meat-inspection.

"The cysticercus cellulosæ has, up till now, been found in the various parts of different muscles, especially in those of the heart, in the cellular tissue, in the brain, and in the eye of man. In all these places, according to the space it has for development, the cyst. cell. assumes a different form and size. Its most striking forms are found in the lobes of the brain and in the eye. The softness and looseness of the tissue determines the size and form of the cysticercus."

It may be of interest to say, briefly, that many attempts have been made to prove experimentally the transformation of the eggs of the tænia solium into the cysticercus. Dr. von Ammon and Prof. Heubner made this experiment by placing the eggs of the tænia solium into the conjunctiva, which had been previously opened for the purpose. These experiments were without result.

Up till now little attention has, alas, been given to the appearance of cyst. cell. in the eye of animals. Text-books contain only meagre references, possibly owing to the fact that the disease is so rare.

During three months of last year in the slaughter-house here I examined 400 swine affected in different degrees with cyst. cell. on the muscles of the eye, the eyelids, and the inner side of the eyeball. The result of this investigation proved again that the occurrence of cyst. cell. in the eye of swine is very seldom, and a still greater rarity on the eyelids and eye-muscles. Of the 400 cases pustules were found inside the eye only twice, beneath the retina. Twenty per cent. showed an affection of the eye muscles, but only when the animal was severely affected and the masticatory muscles, those of the neck and foreparts, were saturated with pustules.

In seventeen cases I found the pimples on the eyelids close to the inner corner of the eye. The two cases of subretinal cysticercus were found in swine which were otherwise little affected, and it is very possible that swine which are not recognized as infected at the time of inspection may show this trouble in the eye. It is therefore worthy of a closer examination in the future.—*Thierarzt. Centralblatt*, June 1, 1898.

CHEAP PRACTITIONERS. The *Brunn Volkszeitung*, under the headline, "A Beautiful Help for Cattle," relates the following incident: "In the community of Lichnau, on May 14th, Franz Petr summoned the horse farrier of his native place to visit a mare suffering from colic. The farrier declared that the mare was about to give birth to a colt. Not being able to gain access to the foal, he pulled out his knife, cut a hole in the vagina, and out of this hole pulled a piece of intestine a yard long which he declared to be the placenta. The horse died, naturally, after such a performance, the loss amounting to a hundred dollars."

What have the zealous advocates of "cheap practitioners" to say to this?—*Idem.*

Development of the Embryo of the Horse. According to Prof. Ewart, in the horse transition from the nourishment by the amnion (yolk-sack) to that of the allantoid takes place between the sixth and ninth week. The embryo is now connected at one pole more directly with the wall of the uterus. The offshoots of the allantoid during these eight weeks are still small, and the fixation of the embryo is not firm, so that at this time, in reference to nourishment, it is passing a critical point. Besides this, sexual excitation takes place in the third, sixth, and ninth weeks, and this can become dangerous to the embryo. For this reason abortion in mares frequently takes place during the sixth to ninth week of pregnancy. As a means of prevention, Ewart recommends that all changes in food, temperature, stall, and surroundings be avoided.—*Idem.*

A Test of Fresh Milk and the Impurities Contained Therein. According to the *Rundschau fur Pharmacie, Chemie, Hygiene*, etc., Vaudin recurs again to the well-known phenomenon that milk which has been colored pale-blue with a few drops of indigo solution, after a short time loses this color. This phenomenon which, by an infusion of air can be repeated many times anew, is due, according to Duchalaux, to the activity of baceteria contained in the milk. In fact, boiled milk in which the microörganisms have been killed does not manifest this phenomenon. The purer and fresher the milk the longer it remains discolored, until all the effects of the indigo solution vanish. For a long time the freshness of milk has been tested by the fact whether it would stand boiling without coagulation. Wholly aside from the fact that many dairies which stand this test may be pretty old, it is well known that coagulation from boiling can be prevented by the addition of natrium bicarbonate or borax. In regard to the reaction with indigo solution, these means are without effect and cannot hide the truth. According to Vaudin, this test is therefore especially well fitted to keep old milk out of the market. A simple regulation could be enacted that all milk offered for sale must retain the discoloration from indigo solution for a certain time. This period of time would have to be determined by careful experience and, since it depends upon temperature, also for different temperatures. According to the author, the time for temperatures below 15° C. is at least twelve hours; for 15° to 20° about eight hours, and for temperatures above 20° C. four hours. As a result of such a regulation, Vaudin expects greater cleanliness in milking on the part of the farmers and the preservation of lower temperatures in storing and transportation.—*Idem.*

ORGANO-THERAPEUTICS. Landau recommends that organo-therapeutics should be opposed for the present on the ground of lack of proof. The whole basis of this therapeutics is very doubtful, for even if certain diseases are caused by disturbances of the glands, in most cases it is a question of the failure of the function, which cannot be replaced by dead tissue dissolved and chemically changed by digestion, because function is an activity of living, not dead cells.—*Idem.*

